# Bifid T waves in leads V2 and V3 in children: a normal variant

**DOI:** 10.1186/1824-7288-35-17

**Published:** 2009-06-26

**Authors:** Maria Pia Calabrò, Ignazio Barberi, Antonella La Mazza, Maria Chiara Todaro, Francesco L De Luca, Lilia Oreto, Mario Salvatore Russo, Marco Cerrito, Letteria Bruno, Giuseppe Oreto

**Affiliations:** 1 Department of Paediatrics, University of Messina, Messina, Italy; 2 Department of Medicine and Pharmacology, University of Messina, Messina, Italy

## Abstract

**Introduction:**

The T wave is rarely bifid, apart from patients with long QT syndrome or subjects treated with antiarrhythmic drugs. At times, a U wave partially superimposed upon the T wave is responsible for an apparently bifid T wave. Bifid T waves, in contrast, have been described in normal children in the past, but the phenomenon has not received any attention in recent years, to the extent that it is not mentioned in current textbooks of paediatric cardiology. Aim of the present study was to determine the incidence and clinical counterpart of bifid T waves in a paediatric population.

**Methods:**

We selected 604 consecutive children free from clinically detectable heart disease; subjects whose electrocardiogram showed a bifid T wave underwent a complete clinical and echocardiographic examination. In addition, the electrocardiograms of 110 consecutive adults have also been analyzed. A T wave was considered as bifid whenever it was notched, being the 2 peaks separated from each other by a notch with duration ≥ 0.02 sec and voltage ≥ 0.05 mV. Moreover, in 7 children with bifid T wave in lead V2 further precordial recordings were obtained: a small electrode was gradually moved from V1 to V3, and 4 additional leads were recorded: 2 between V1 an V2, and 2 between V2 and V3.

**Results:**

A bifid T wave was observed in 110 children (18,3%), with a relatively age-related incidence; the highest rate of bifid T waves (53%) occurred in the group of 5-year-old children. The bifid T wave was detected only in lead V2 in 51 cases (46,4%), only in lead V3 in 5 cases (4,6%), in both leads V2 and V3 in 50 cases (45,4%), and in leads other than V2 and V3 in 4 cases (3,6%). In the adult group, none of the examined electrocardiograms showed bifid T waves in any lead.

In the bifid T wave paediatric population, the echocardiogram did not reveal any abnormality, apart from 3 subjects which had an asymptomatic mitral valve prolapse; a trivial mitral and/or tricuspid regurgitation detected by color Doppler, as well as a patent foramen ovale in infants, were not considered as abnormal findings. The QTc interval was normal in all of the subjects; the average QTc interval was not different in the bifid T wave population (402 ± 46 msec) with respect to the control group (407 ± 39 msec).

**Conclusion:**

The incidence of bifid T waves in leads V2 and V3 in normal children is high, and awareness of this phenomenon avoids possible misinterpretations leading to a diagnosis of ECG abnormalities.

## Introduction

The T wave is variable in shape under physiological or pathological conditions, but rarely shows a bifid configuration in adults; this phenomenon can be observed in patients with some forms of long QT syndrome [1], in subjects taking Class 3 antiarrhythmic drugs, particularly Amiodarone [2], in patients with alcoholic cardiomyopathy [3,4] or in the presence of central nervous system lesions [5]. At times, a U wave partially superimposed upon the T wave is responsible for an apparently bifid T wave: in such a situation, often associated with electrolyte imbalance [6], the second wave hump is a U wave rather than part of the T wave.

Bifid T waves, in contrast, have been described in the past in normal children [7-9], but the phenomenon has not been emphasized in recent years, to the extent that it is not mentioned in current textbooks of paediatric electrocardiography. The present study was aimed at exploring the incidence and clinical counterpart of bifid T waves in children, namely whether they are merely a normal variant or can be associated with any disease.

## Methods

At the cardiology outpatient clinic of our Paediatric Department, 604 consecutive children were selected from March to May 2008, on the basis of the following: 1) absence of clinically detectable heart disease; 2) good quality electrocardiogram (ECG), with at least 4 consecutive QRS complexes in each lead; 3) absence of ECG abnormalities such as arrhythmias (apart from respiratory sinus arrhythmia), bundle branch block, atrial enlargement, ventricular hypertrophy, preexcitation.

Whenever a bifid T wave was detected, a complete clinical and echocardiographic examination was performed using an Acuson Sequoia machine.

The ECGs of 110 consecutive adult healthy subjects observed at the cardiology outpatient clinic of the Department of Medicine and Pharmacology, have also been examined.

In any ECG, a search was done for the presence of bifid T waves; in addition, the heart rate and the QTc interval were measured. A T wave was defined as bifid whenever it was notched, being the 2 peaks separated from each other by a notch with duration ≥ 0.02 sec and voltage ≥ 0.05 mV.

Moreover, in 7 consecutive children with bifid T wave in lead V2 further precordial recordings were obtained; a small electrode was gradually moved from V1 to V3, and 4 additional leads were recorded: 2 between V1 an V2, and 2 between V2 and V3.

## Results

**Figure 1:**
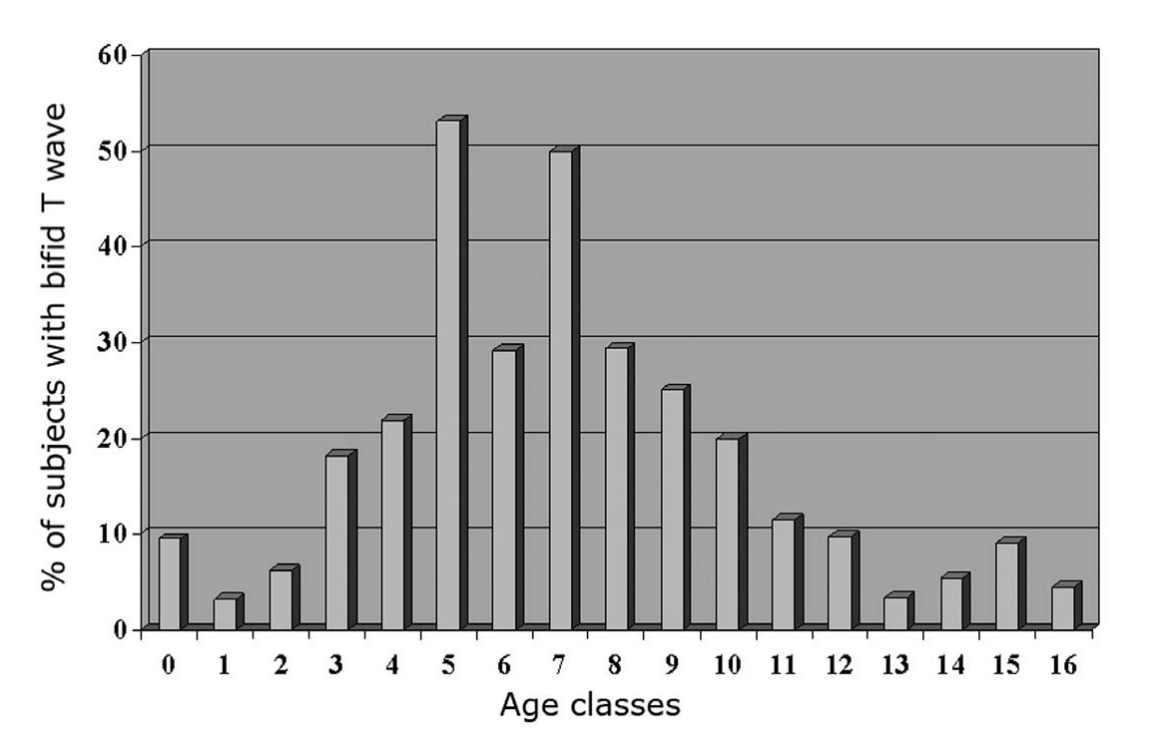
**Incidence of bifid T wave in the age classes**.

**Figure 2:**
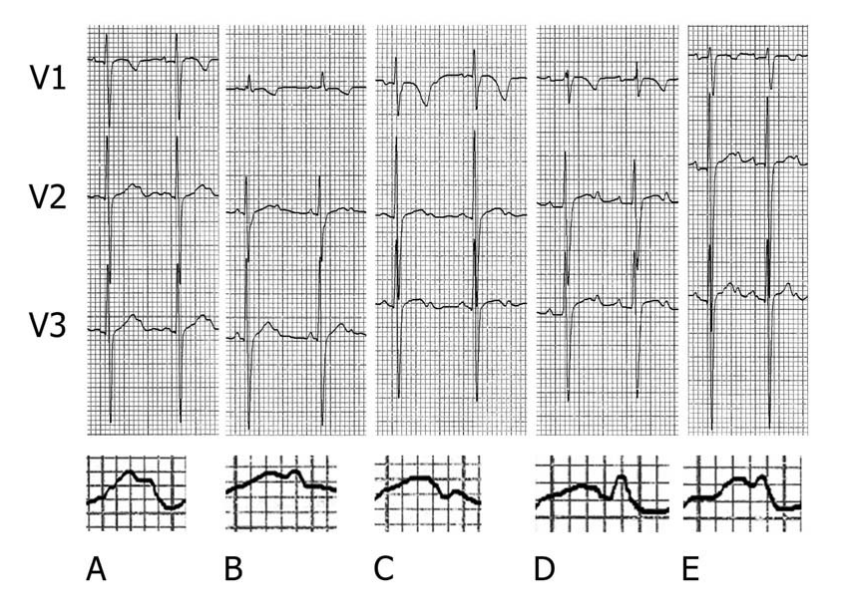
**Elettrocardiograms (leads V1–V3) recorded in 5 healthy children**. The bottom row shows an magnification of the T wave in lead V2.

The mean age of the 604 children was 7.3 ± 3.2 years (range 3 months-16 years). The subjects were divided in 17 groups, each corresponding to 1 year of age. A bifid T wave was observed in 110 children (18,3%), with a relatively age-related incidence; Figure 1 reflects the prevalence of bifid T wave in the age classes: the phenomenon is relatively rare in infants and in teen-agers, whereas the highest rate of bifid T wave (53%) occurs in the 5-year-old children group. Examples of bifid T waves are shown in Figure 2; in some cases the T wave has a "dome and dart" configuration, with a second component peaked and clearly separated from the first one (panels D, E).

The bifid T wave was detected only in lead V2 in 51 cases (46,4%), only in lead V3 in 5 cases (4,6%), in both leads V2 and V3 in 50 cases (45,4%), and in leads other than V2 and V3 in 4 cases (3,6%). In the adult group, none of the examined ECGs showed bifid T waves in any lead.

**Figure 3:**
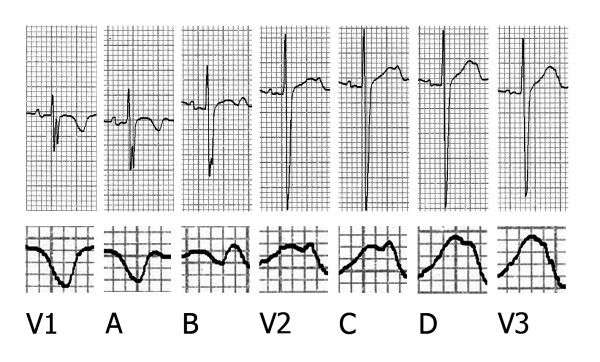
**Precordial recordings obtained displacing gradually the electrode from V1 to V3**. A e B are recordings intermediate between V1 and V2; C e D are intermediate between V2 and V3.

The additional leads recorded in 7 subjects (2 recordings between V1 an V2, as well as between V2 and V3) showed always the pattern represented in Figure 3. The T wave was totally negative in lead V1, but became less negative, with a terminal minimally positive deflection, when the electrode was slightly displaced towards the left (panel A). A further displacement of the electrode in the same direction (panel B) resulted in reduction of the negative T wave component and increase of the positive part, so that a bifid T wave appeared. Lead V2, in turn, showed again a typical bifid T wave, and the same morphology was once more recorded with the electrode minimally shifted leftward (panel C); a further displacement (panel D) resulted in increase of the first positive component of the T wave, and finally V3 showed either a bifid or an entirely positive T wave (panel E).

In the bifid T wave population, the echocardiogram did not reveal any abnormality, apart from 3 subjects which had an asymptomatic mitral valve prolapse; a trivial mitral and/or tricuspid regurgitation detected by color Doppler, as well as a patent foramen ovale in infants, were not considered as abnormal findings.

A U wave clearly separated from the bifid T wave (Figure 2, panels A and B) has been detected in leads V2 and/or V3 in 31 children (28%). The QTc interval was normal in all of the subjects; the average QTc interval was not different in the bifid T wave population (402 ± 46 msec) with respect to the control group (407 ± 39 msec).

## Discussion

The reported data show that a bifid T wave in leads V2 and V3 is a normal pattern in children, particularly in those aged 5 to 8 years; in healthy adults, in contrast, no bifid T wave is detectable in any lead. The statement that a bifid T wave in leads V2 and V3 is a normal finding in children is reinforced by the absence of detectable heart disease in all of our subjects; in addition, the normal QTc interval denies that such a phenomenon expresses a repolarization abnormality.

### Origin of the bifid T wave

The bifid T wave was present only in leads V2 and V3, and never in the limb leads or in leads V1, V5 or V6. This finding suggests that the phenomenon depends on the peculiar orientation of the electrical forces generated by ventricular repolarization on the horizontal plane.

**Figure 4:**
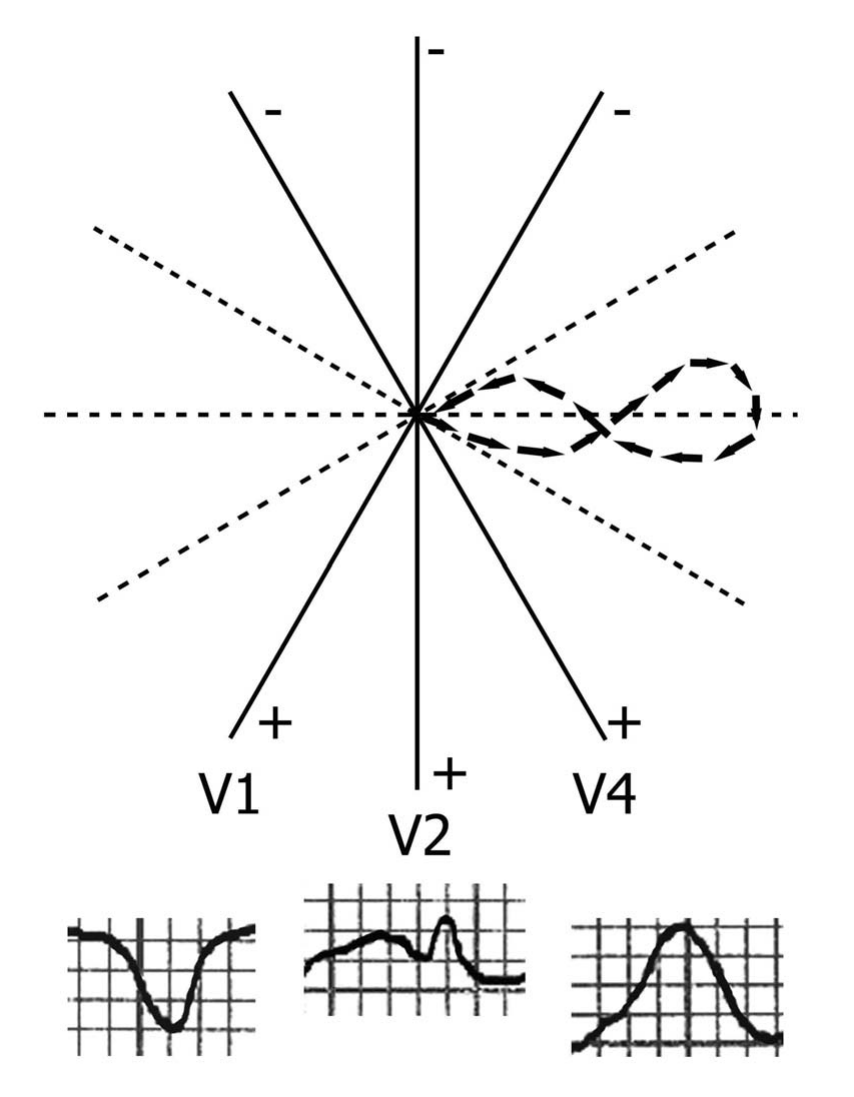
**Genesis of the bifid T wave**. Vectorcardiographic T loop on the horizontal plane; T waves recorded in V1, V2, and V4.

In order to point out the mechanism underlying the bifid T wave in V2 and V3, we obtained in 7 subjects additional recordings by moving the electrode from V1 to V3. A progressive T wave change from a negative to a positive shape was always observed, and the T wave showed a bifid configuration during transition from negativity to positivity. This observation has led us to the following explanation, represented in Figure 4.

In subjects with bifid T wave, the T wave vectorcardiographic loop in the horizontal plane shows a figure-of-eight shape with rotation initially counterclockwise and subsequently clockwise: the first vectors are directed leftward and slightly anteriorly, and then the electrical forces move posteriorly. The second part of the loop rotates in a clockwise direction, being displaced anteriorly, and only the final section of the loop is again counterclockwise with the terminal forces directed posteriorly.

In accordance with this sequence of electrical forces, the T wave is negative in lead V1 because all the vectors project on the negative part of the lead line. Lead V2, whose positive pole is at +90°, in turn, "sees" as positive the first T wave vectors, projecting on the positive part of the lead line, while the ensuing vectors, directed posteriorly, are "seen" as negative. When the late section of the loop is again displaced anteriorly, a second T wave positivity occurs.

The two T wave positive components in lead V2, thus, are due to the 2 groups of anterior forces, whereas the intermediate notch expresses the vectors directed posteriorly. When the recording electrode is displaced towards the left, the amount of vectors projecting on the positive part of the lead line increases, and the notch between the two cusps becomes less and less pronounced until the T waves becomes entirely positive, as it happens in lead V4, whose positive pole is at +60°.

### Clinical relevance of the bifid T wave

The incidence of bifid T waves in leads V2 and V3 in normal children is high, and awareness of this phenomenon avoids possible misinterpretations leading to a diagnosis of ECG abnormalities.

The first diagnostic problem, in the presence of a bifid T wave, is distinction from a U wave more or less coinciding with the T wave. Some drugs, particularly antiarrhythmic drugs, as well electrolyte imbalance such as hypokalemia, prolong the QT interval and give rise to a prominent U wave that is partially superimposed upon the T wave, simulating a bifid morphology of this. Drug or hypokalemia-induced QT prolongation and related T wave change, however, are not limited to leads V2 and V3 but occur in several leads, including the limb leads. In our normal children, in contrast, bifid T waves have been observed only in V2 or V2 and V3; in addition, the simultaneous presence of bifid T wave and U wave, observed in several cases, rules out the possibility that the second "hump" of the bifid T wave was indeed a U wave.

Another possible abnormal condition simulated by the bifid T wave is a non-conducted P wave superimposed upon the T wave and responsible for the bifid configuration: in our series, however, no atrial extrasystoles were evident in any lead, and furthermore no bifid T wave was followed by a pause, as it generally occurs in non-conducted atrial extrasystoles. Accordingly, this hypothesis was ruled out.

A bifid T wave in leads V2 and/or V3 should be considered a normal phenomenon in children; the absence of this pattern in adults demonstrates that the ventricular repolarization process changes with aging, in such a way that the T loop, that has a figure-of-eight shape in very young subjects, becomes entirely counterclockwise in adults, as it normally occurs [10].

### Study limitations

A vectorcardiogram would have provided a definite demonstration of the relationship between a figure-of-eight T loop in the horizontal plane and the bifid T wave in leads V2 and V3. In our Institution, however, no vectorcardiographic machine was available, and no vectorcardiographic examination was performed.

## Competing interests

The authors declare that they have no competing interests.

## Authors' contributions

MPC carried out the clinical examination of subjects, and coordinated the study. IB participated in data analysis and drafted the manuscript. ALM recorded the ECG tracings in the paediatric population, particularly those with additional precordial leads. MCT carried out the ECG analysis. FLDL participated in the design of the study. LO contributed in analyzing the Electrocardiograms. MSR performed the echocardiograms. MC recorded and analyzed the electrocardiograms in the adult population. LB performed the Echocardiograms. GO conceived of the study, participating in its design and coordination, and revised the manuscript. All authors read and approved the final manuscript.
